# A New Ligature

**Published:** 1904-03-12

**Authors:** 


### A NEW LIGATURE.

IN the letter which was published under the above title in our issue of the 27th ult. the writer's name should have appeared as Mr. P. H. V. Hammersley, and not as it was printed.

### "TORFIT" (William E. Farrer, Star Works, Cambridge Street, Birmingham).

THE manufacturers of this material have requested us to state, in view of a rumour which has been circulated to the effect that the material is protected by an English patent, that the only English application for patent for this class of material that has been made is dated later than the period when they first commenced to sell "Torfit," and consequently cannot have any bearing whatever on that material.

